# National UK Survey of Radiation Doses During Endovascular Aortic Interventions

**DOI:** 10.1007/s00270-023-03592-x

**Published:** 2023-11-15

**Authors:** Yvonne Tsitsiou, Bar Velan, Rebecca Ross, Raghu Lakshminarayan, Andy Rogers, Mohamad Hamady, RADEVAIR, Lamran Khan, Ananth Krishnan, Martin Hennessy, Ram Kasthuri, Zenaib Al-Rekabi, Said Abisi, Mark Hampshire, Panos Goutzios, Muhammad Hanif, Emma Olivier, Andrew Wood, Andrew Macey, Sachin Modi, Robert Allison, Clare Bent, Peter Bungay, Robert Whiteman, Robin Williams, Zaid Aldin, Josephine Weaver, Robert Kaikini, David Wells, John Hancock, Anil Madhavan, Sapna Puppala, Matthew Matson, Katharine Lewis, Raman Uberoi, Andrew Winterbottom, Bella Huasen, Michael Jenkins, Trevor Cleveland, Rachel Butcher

**Affiliations:** 1 Imperial College Healthcare NHS Trust, St. Mary’s Hospital, Praed St, London, W2 1NY UK; 2 Department of Surgery and Cancer, Imperial College London, London, UK; 3 Hull University Teaching Hospitals NHS Trust, Hull, UK; 4 Nottingham University Hospitals NHS Trust, Nottingham, UK

**Keywords:** Diagnostic reference level, Endovascular aortic aneurysm repair, Radiation protection, Aorta, Radiation dose

## Abstract

**Purpose:**

Endovascular aortic repair (EAR) interventions, endovascular abdominal aortic repair (EVAR) and thoracic endovascular aortic repair (TEVAR), are associated with significant radiation exposures. We aimed to investigate the radiation doses from real-world practice and propose diagnostic reference level (DRL) for the UK.

**Materials and Methods:**

Radiation data and essential demographics were retrospectively collected from 24 vascular and interventional radiology centres in the UK for all patients undergoing EAR—standard EVAR or complex, branched/fenestrated (BEVAR/FEVAR), and TEVAR—between 2018 and 2021. The data set was further categorised according to X-ray unit type, either fixed or mobile. The proposed national DRL is the 75th percentile of the collective medians for procedure KAP (kerma area product), cumulative air kerma (CAK), fluoroscopy KAP and CAK.

**Results:**

Data from 3712 endovascular aortic procedures were collected, including 2062 cases were standard EVAR, 906 cases of BEVAR/FEVAR and 509 cases of TEVAR. The majority of endovascular procedures (3477/3712) were performed on fixed X-ray units. The proposed DRL for KAP was 162 Gy cm^2^, 175 Gy cm^2^ and 266 Gy cm^2^ for standard EVAR, TEVAR and BEVAR/FEVAR, respectively.

**Conclusion:**

The development of DRLs is pertinent to EAR procedures as the first step to optimise the radiation risks to patients and staff while maintaining the highest patient care and paving the way for steps to reduce radiation exposures.

## Introduction

Endovascular aneurysm repair (EAR) interventions—endovascular abdominal aortic repair (EVAR) and thoracic endovascular aortic repair (TEVAR)—have become routine practice owing to their minimally invasive nature, shorter admission times and a lower rate of short-term complications compared to open repair [1].

Due to the potentially complex nature of EAR interventions, high radiation doses can be expected [2–4]. Therefore, optimising the dose-image quality balance is key for staff and patient safety. To aid the process of optimisation, the International Commission on Radiological Protection (ICRP) has developed the term diagnostic reference level (DRL) [5]. This is a value of procedure dose to the patient, normally expressed as both a kerma area product (KAP) and fluoroscopy time, determined from median values at a wide range of facilities, that seeks to guide centres in understanding whether their doses are high and if so, investigate if patient doses may be lowered. Most radiological examinations have nationally agreed DRLs to guide optimisation. However, there is a lack of DRLs for interventional procedures in the UK and other European countries.

The following confounding factors should be considered to develop DRLs relevant to aortic interventions. The term EAR covers a wide range of interventions, from standard EVARs to more complex fenestrated endovascular abdominal aortic repair (FEVAR), branched endovascular aortic repair (BEVAR) and TEVAR interventions. Furthermore, the manufacturer, model, type (fixed vs mobile theatre C-arm) and age of imaging equipment play key roles in determining the patient dose. Tuthill and colleagues proposed an interim European DRL [6], and a local DRL was proposed in European countries such as Spain and Greece [4, 7]. The UK does not presently have a nationally accepted DRL for such interventions. This realisation drove us to recruit centres across the UK to survey current dose levels and propose national DRLs.

In this national survey, we aimed to provide national DRLs so individual departments may optimise their practices and provide lower radiation exposure to patients and staff and discuss the differences in doses due to differing subcategories of EAR interventions and the X-ray equipment used.

## Materials and Methods

This retrospective study contains data collected from joint interventional and vascular centres in the UK for all patients undergoing EVAR (standard or branched/fenestrated) and TEVAR between 2018 and 2021. Centres included a range of regional teaching hospitals and local general hospitals, located in England, Wales and Scotland. Ethical approval for this retrospective study was waived according to institutional guidelines. Due to the study’s retrospective, anonymised, noninterventional nature based solely on data generated and documented during clinical practice, informed and written consent was not required.

In total, 27 out of the 67 Interventional and Vascular Surgery centres in the UK, according to the National Vascular Registry (NVR), were emailed requesting data. The data requested included age, sex, type of procedure and date, X-ray unit model, procedure KAP, fluoroscopy time, cumulative air kerma (CAK) and fluoroscopy KAP and CAK. Data were collected from electronic patient notes, picture archiving and communication systems (PACS) and angiographic systems, as well as patient dose management systems where available.

The data set was divided into standard EVAR, FEVAR/B EVAR and TEVAR and further categorised according to X-ray unit type (i.e. fixed or mobile). Each X-ray unit was analysed separately to form the distribution of device medians. All data provided, irrespective of procedure outcome, were included in statistical analysis, which was carried out using SPSS Statistics 27. Normality tests were performed, and medians and boxplots were used because the data were nonparametric. The proposed national DRL is the 75th percentile of the collected medians [5], of centres with five or more cases. The data were presented in graphs in the order of ascending medians, and the centres’ names were anonymised into numbers.

## Results

Data from 3712 endovascular aortic procedures were collected from 24 centres, out of which 6 were local general hospitals and 18 were regional teaching hospitals. The mean age was 74.6 (± 10.0 SD) years old, and 84.2% were males and 15.8% were females. The majority of endovascular procedures, 3477/3712, were performed on fixed X-ray units, and 235/3712 were performed on mobile units. Of those performed on fixed units, 2062 cases were standard EVAR, 906 were BEVAR/FEVAR, and 509 were TEVAR. Of those performed on mobile units, 129 were standard EVAR, 106 cases were BEVAR/FEVAR, and 26 were TEVAR. All data provided underwent a quality assurance review, where if any centres provided data that were outside of expected values, they were emailed to clarify the units provided. Due to the retrospective nature of the study, some data sets were incomplete; however, all data were further analysed.

**Fig. 1:**
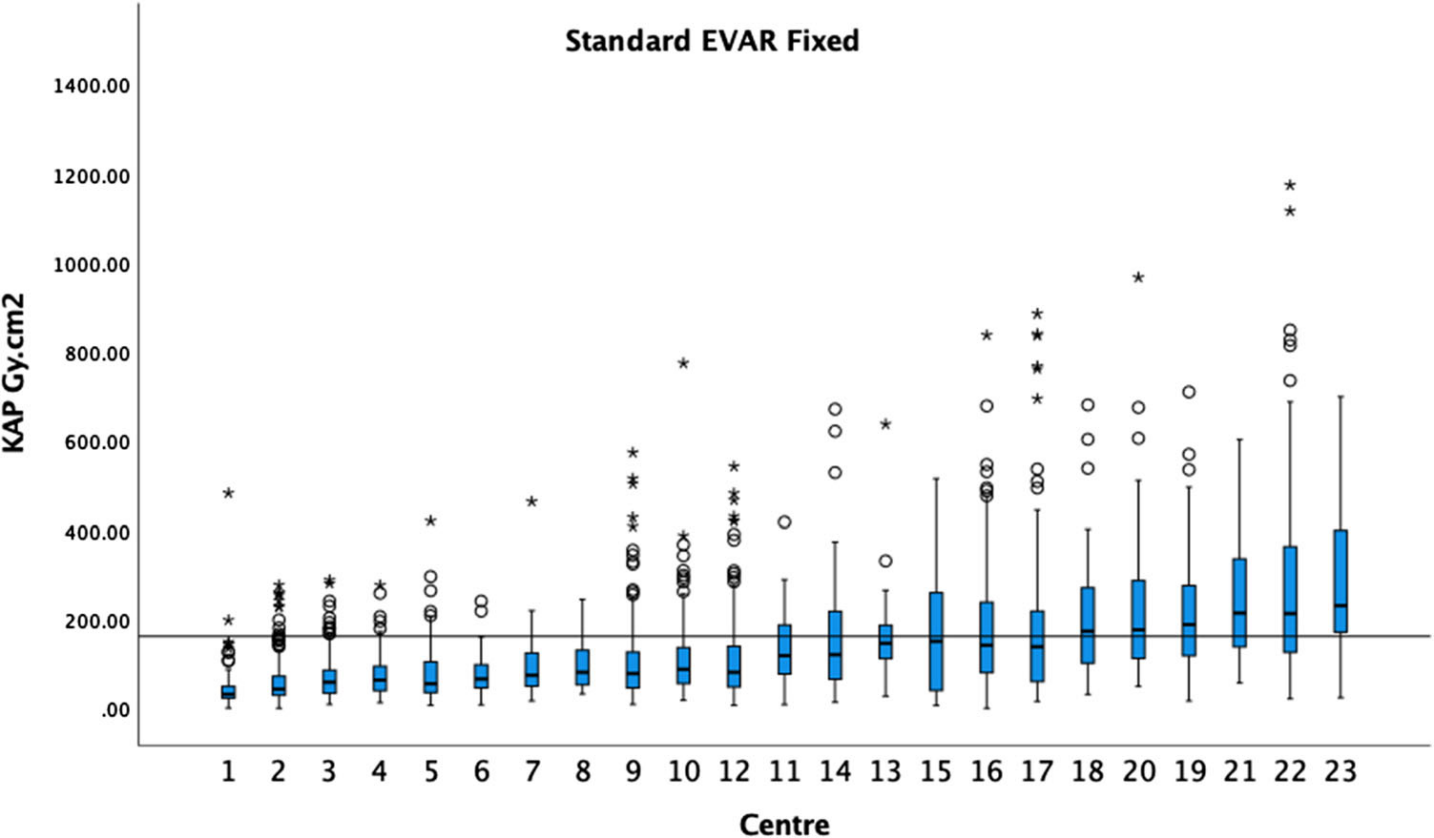
The median KAP and boxplots of standard EVAR on fixed X-ray units in ascending order. The 75th percentile of the medians was 162 Gy cm^2^ as demonstrated by the black horizontal line. Circles indicate outliers and asterisks extreme outliers in all graphs

**Fig. 2:**
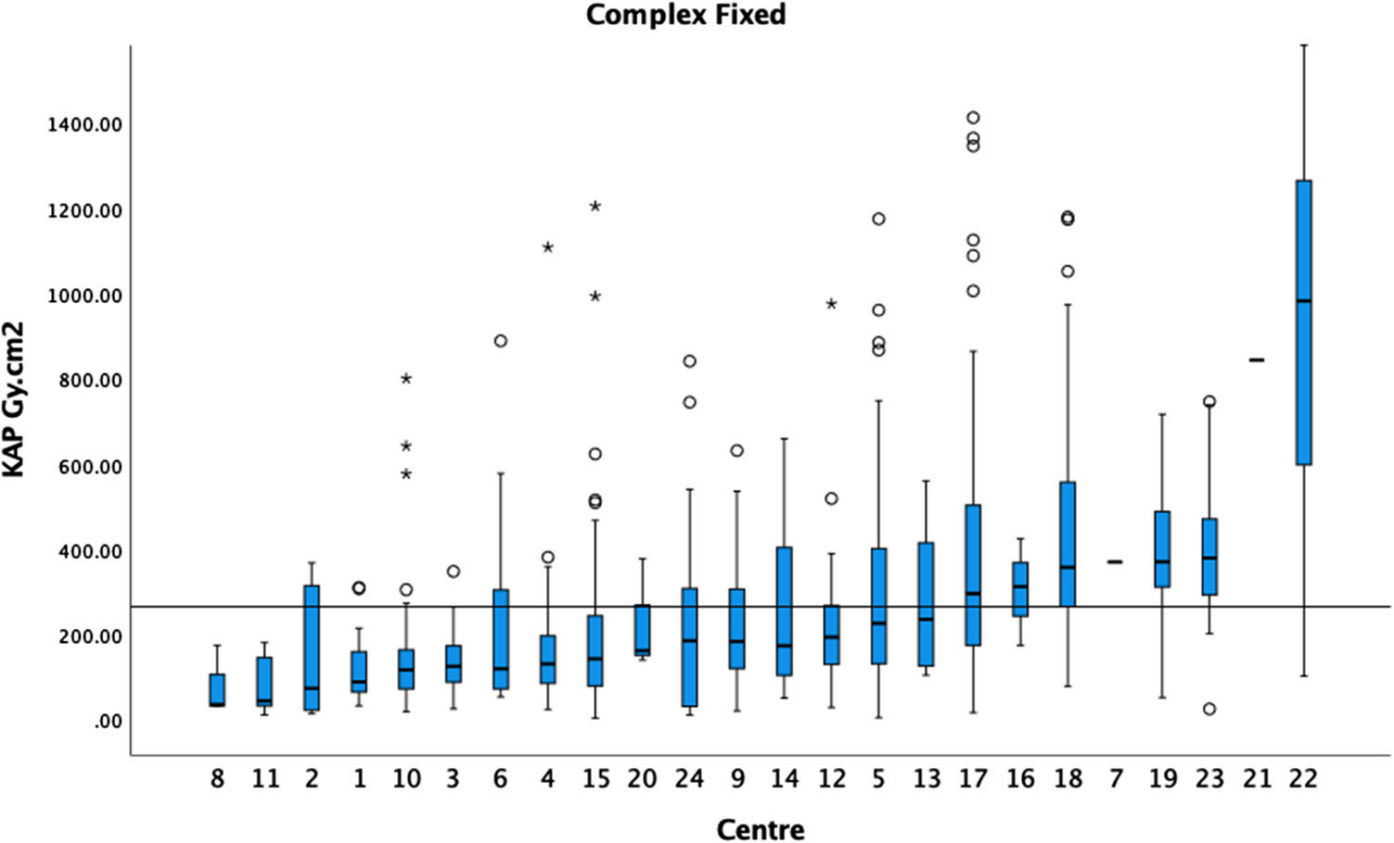
The median KAP and boxplots of BEVAR/FEVAR on fixed X-ray units in ascending order. The 75th percentile of the medians was 266 Gy cm^2^ as demonstrated by the black horizontal line. Centres with fewer than 5 cases are included in this graph

**Fig. 3:**
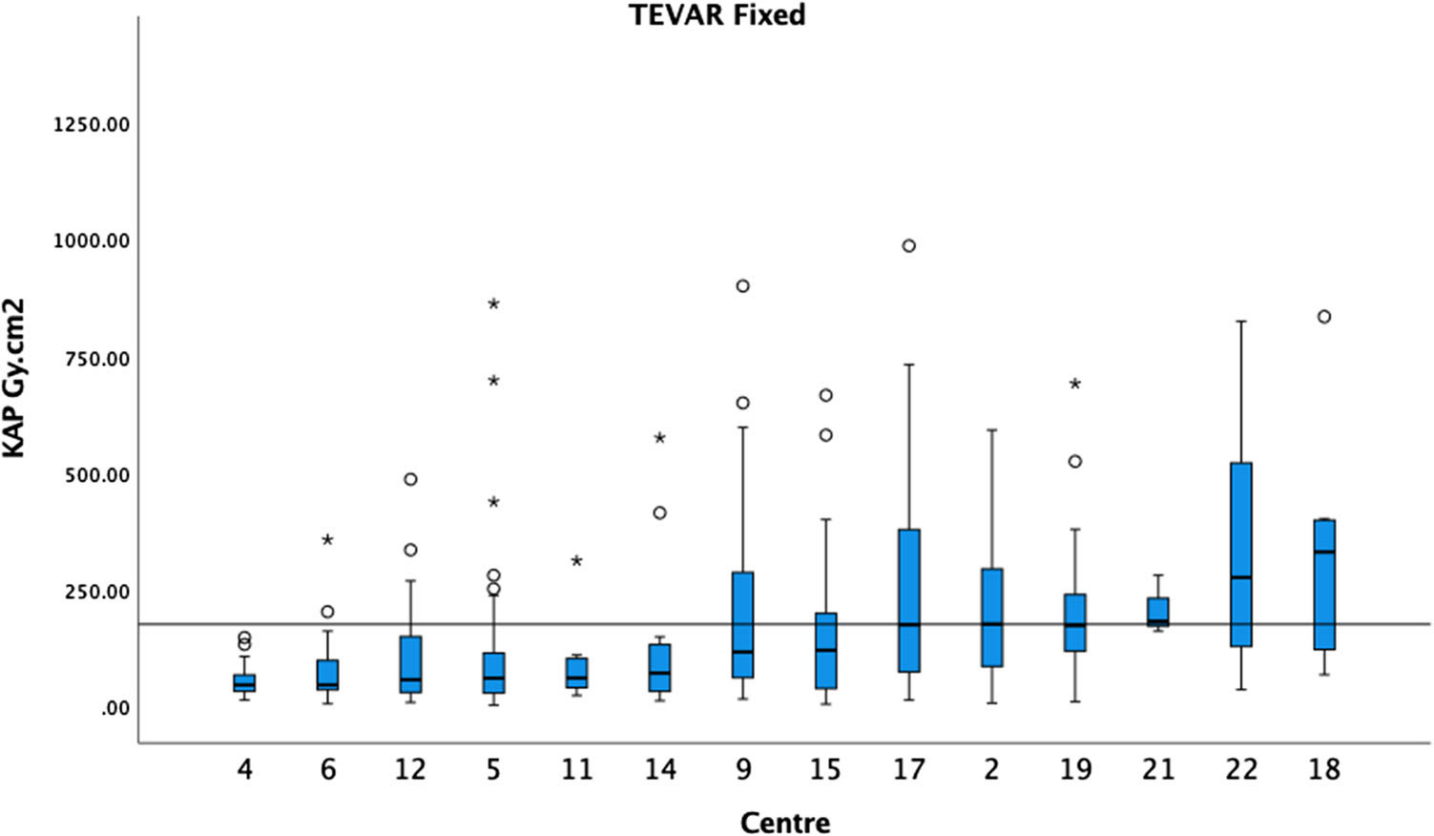
The median KAP and boxplots of TEVAR on fixed X-ray units in ascending order. The 75th percentile of the medians was 175 Gy cm^2^ as demonstrated by the black horizontal line

Figure 1 demonstrates the KAP medians and boxplots performed on fixed machines for standard EVAR procedures in ascending order. The 75th percentile of the medians was 162 Gy cm^2^. Figure 2 demonstrates the KAP medians and boxplots of complex endovascular procedures, BEVAR and FEVAR, performed on fixed machines. The 75th percentile of the medians was 266 Gy cm^2^ (5 centres excluded). Figure 3 demonstrates the KAP medians and boxplots of TEVAR procedures on fixed machines. The 75th percentile of the medians was 175 Gy cm^2^ (1 centres excluded) (Fig. 3).

**Fig. 4:**
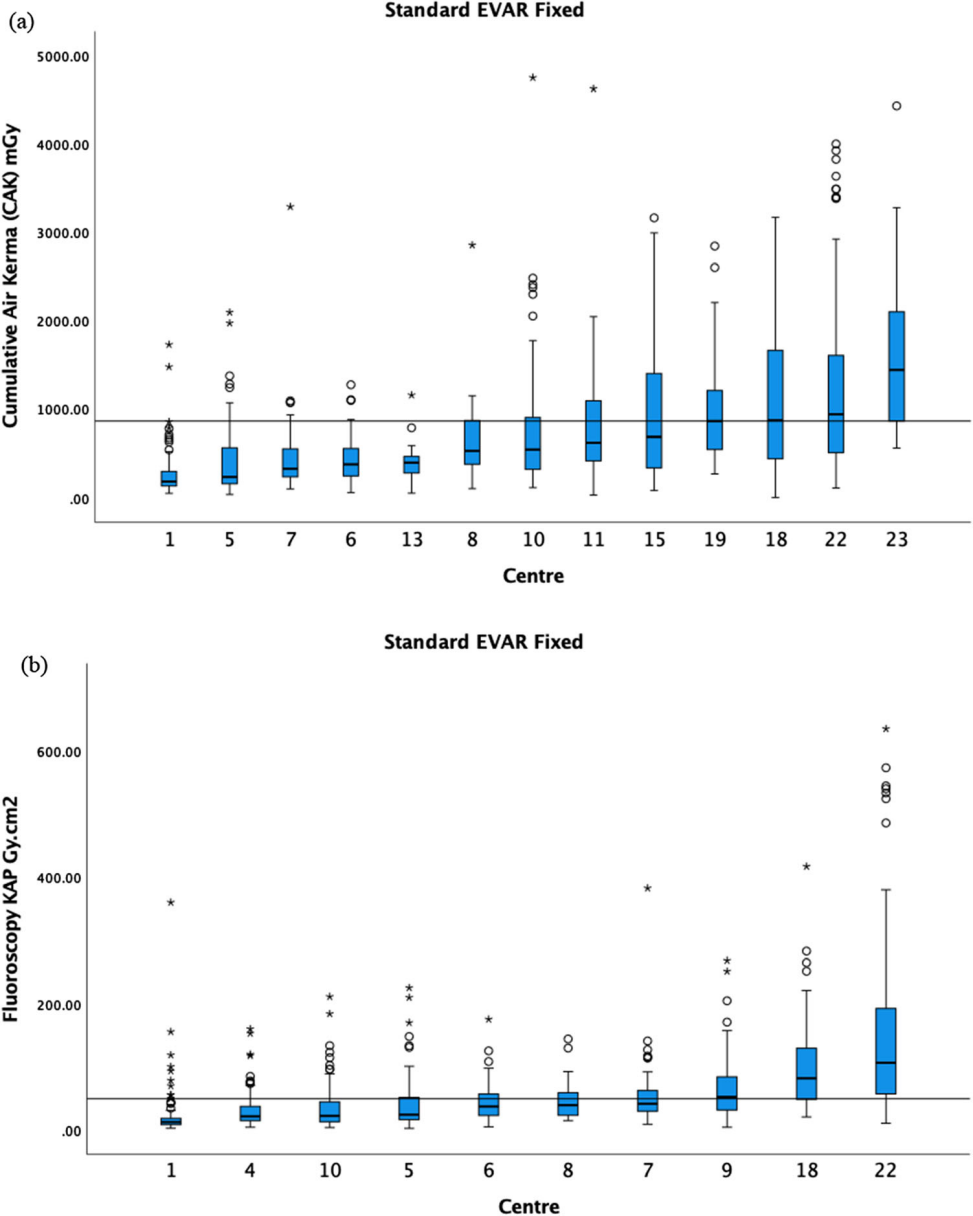
**a** The median CAK and boxplots of standard EVAR on fixed X-ray units in ascending order. The 75th percentile of the medians was 865 mGy as demonstrated by the black horizontal line. **b** The median fluoroscopy KAP and boxplots of standard EVAR on fixed X-ray units in ascending order. The 75th percentile of the medians was 49 Gy cm^2^ as demonstrated by the black horizontal line

**Fig. 5:**
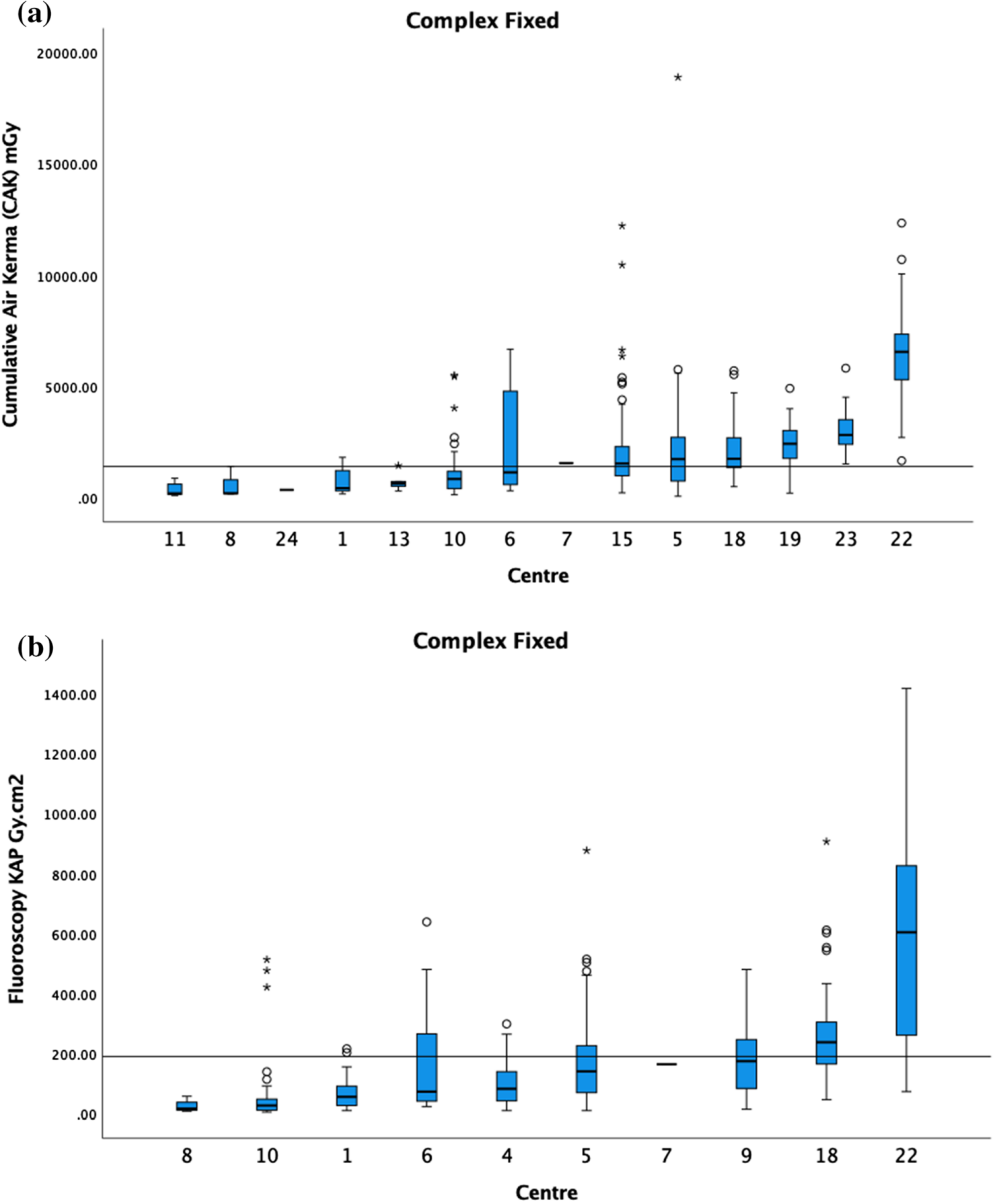
**a** The median CAK and boxplots of BEVAR/FEVAR on fixed. The 75th percentile of the medians was 1390 mGy as demonstrated by the black horizontal line. Centres with < 5 cases are included in this graph. **b** The median fluoroscopy KAP and boxplots of BEVAR/FEVAR on fixed. The 75th percentile of the medians was 190 Gy cm^2^ as demonstrated by the black horizontal line. Centres with < 5 cases are included in this graph

For standard EVAR, the CAK 75th percentile was 865 mGy, and the fluoroscopy KAP 75th percentile was 49 Gy cm2 (Fig. 4). The fluoroscopy CAK was provided by 6 centres, and therefore, a DRL was not calculated. For BEVAR/FEVAR, the CAK 75th percentile was 1390 mGy (4 centres excluded), and the fluoroscopy KAP 75th percentile was 190 Gy cm^2^ (2 centres excluded) (Fig. 5). The fluoroscopy CAK was provided by 6 centres, ranging from 7 to 213 mGy. For TEVAR, the CAK was reported by 7 centres, ranging from 202 to 1050 mGy; fluoroscopy KAP by 5 centres, ranging from 1.9 to 18.9 Gy cm^2^; and the fluoroscopy CAK by 3 centres, ranging from 8 to 86 mGy. Due to the limited number of centres, no DRL was calculated for the above.

For standard EVAR procedures on mobile X-ray units, only 6 out of the 10 centres providing data on KAP had 5 or more procedures. Similarly, the CAK was only provided by 2 centres. Hence, no national DRL was proposed. Due to the small number of TEVAR cases carried out on mobiles and the majority of BEVAR/FEVAR mobile cases being from a single centre, no national DRL was proposed.

**Fig. 6:**
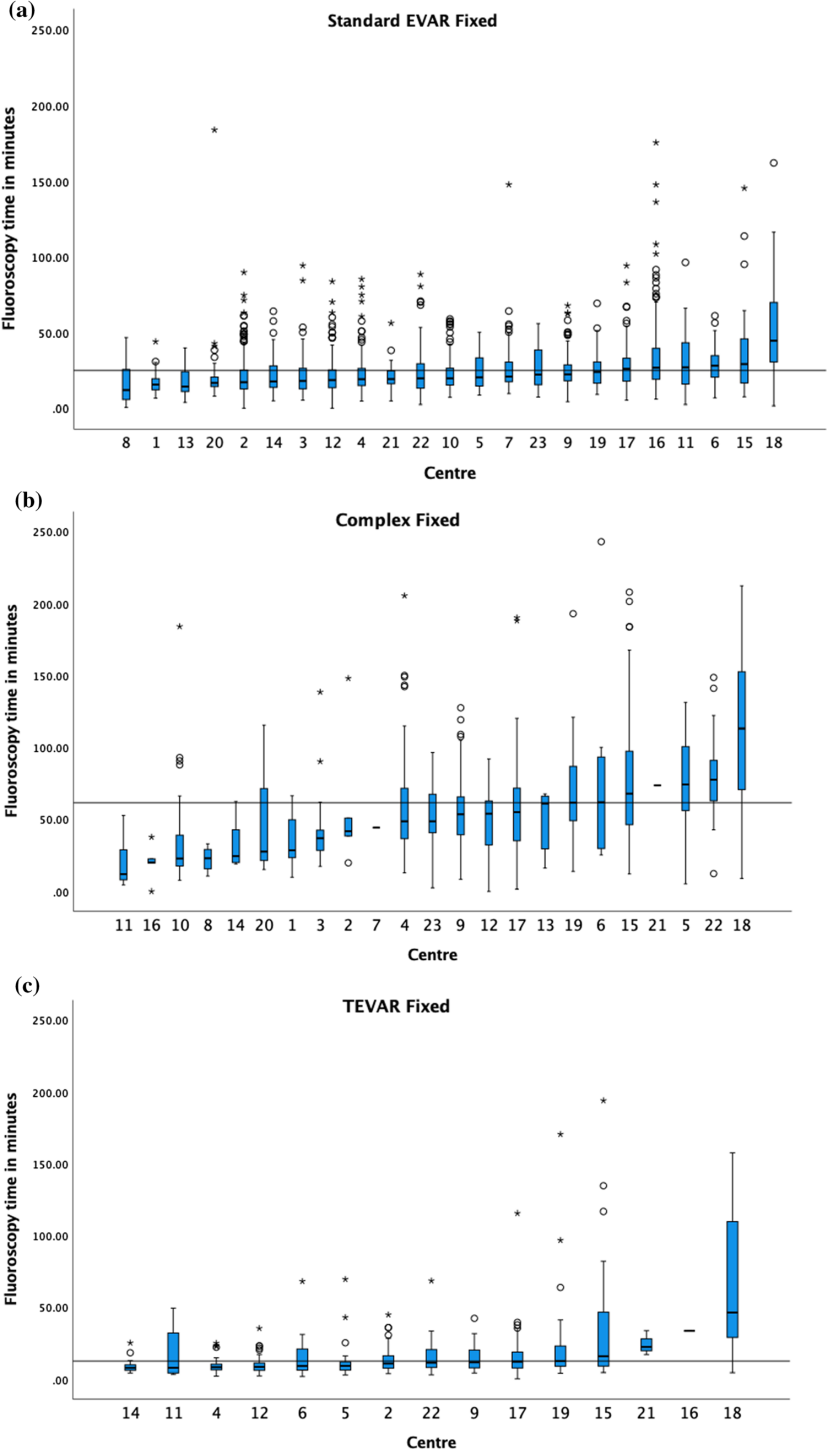
The median fluoroscopy time in minutes and boxplots of **a** standard EVAR, **b** BEVAR/FEVAR and **c** TEVAR on fixed X-ray units, in ascending order. The 75th percentile of the medians was 24.8 min, 61.7 min and 12.1 min as demonstrated by the black horizontal line in each respective graph. Centres with < 5 cases are included in the graphs

The 75th percentile of the medians for the fluoroscopic time in minutes, shown in Fig. 6, for standard EVAR, BEVAR/FEVAR and TEVAR on fixed machines was 24.8, 61.7 (4 centres excluded) and 12.1 min (2 centres excluded), respectively.

## Discussion

With the increasing number of interventional radiology procedures, the population dose also increases [8]. The ICRP and Society of Interventional Radiology recommend the formation and use of DRLs in interventional radiology to improve dose monitoring and management [5, 9, 10]. Specifically, the IRCP (in its publication 135) acknowledges that radiation doses can vary greatly even within the same department for the same procedure, as demonstrated in our study; therefore, implementation can be challenging [5, 11]. Additionally, the European Directive 2013/59/EURATOM requires its member states to establish and utilise DRLs in interventional radiology, with regular reviews [12]. This requirement was transposed into UK regulations by the Ionising Radiation (Medical Exposure) Regulations 2017 [13] and amended in 2018 (UK SI) [14].

Although a DRL is typically defined as the standard dose for a standard-sized patient [5, 12], our data make it clear that doses vary greatly across the UK and within each procedure category. Comparing our data to NVR, patient demographics were similar with 88% and 83% of patients undergoing EVAR and complex being male during 2019–2021, respectively. This study represents close to 40% of NVR cases [15]. Therefore, regular DRL review through a multicentre approach is necessary to keep DRLs updated with ever-advancing technological and clinical practice. Regular audits and reviews will allow local centres to understand their patient doses in a national context to use this information to optimise their procedures, implement up-to-date DRLs and maintain quality assurance. This will help to keep patient doses as low as reasonably achievable (ALARA) [5].

Inter-centre dose discrepancies are multifactorial and may result from nonoptimised fluoroscopic-guided procedures. Nonexhaustive parameters affecting dose include operator experience [16–18], equipment age, dose display with automatic dose monitoring, and further technical factors such as pulse rate, collimation, angulation, magnification, patient size, procedural complexity, state-of-the-art facilities incorporating updated technology and optimised automatically applied settings, all cumulatively reducing the dose [19].

The dose should be minimised while maintaining image quality to allow for optimal patient outcomes. Image fusion (preoperative CT angiography with live fluoroscopy image fusion) has also been extensively shown to reduce the radiation dose [3, 20, 21], contrast volume and procedural time during EVAR [22], with vessel navigation possible without contrast bolus. A few additional factors have been suggested that may also contribute to dose discrepancies (that were not accounted for in our study). One example includes the potential overestimation of KAP due to attenuation by the table and mattress. This may be up to 40% on some systems [23]. However, although KAP displays are often calibrated ‘in air’, one would expect local centres to have systems to adjust these readings to allow for more accurate dose measurement. Furthermore, not all angulations intercept the table, so the amount of overestimation is likely to be less [23]. The latest image-processing techniques that reduce visible noise also allow for significantly lower patient doses [24].

International guidelines suggest patients follow up for skin doses above 5 Gy (CAK) or 500 Gy cm^2^ (KAP) to monitor for radiation-induced skin injury [10]. None of the procedures exceeded 5 Gy for CAK; however, out of the standard EVAR, BEVAR/FEVAR and TEVAR, 2.8%, 14.0% and 5.7% exceeded 500 Gy cm^2^ for KAP on fixed machines, respectively. Patients undergoing endovascular aneurysm repair frequently require follow-up imaging and possible re-intervention, increasing the patients’ annual dose, with endoleaks as a potential complication. Consideration should also be made for the same skin or organ in-field dose being re-irradiated. Centres should have robust procedures in place for the management of high-risk patients and those who exceed trigger levels. Identification of at-risk patients is well described by Jaschke et al. [25] and includes patients who undergo repeated procedures, radiotherapy on the same entry site and weight greater than 120 kg.

Our results show an approximately 3 times reduction in KAP for standard EVAR procedures when carried out on a mobile X-ray unit compared to a fixed X-ray unit. This ratio is comparable with some recent literature [4]. There is quality of care when using mobile X-ray units in aortic interventions; however, this has been criticised by both the Royal College of Radiologists and Medicines and Healthcare products Regulatory Agency (MHRA) [26, 27]. Since few centres still need to use mobile X-ray units for some procedures, we have included the results.

Comparing our data to the proposed interim DRLs by Tuthil and colleagues, the cohort of patients was much smaller, with a total of 180 standard EVAR procedures from 5 centres with similar demographics to this study. Their 75th percentile value KAP was 158.49 Gy cm^2^ for standard EVAR, which is only slightly smaller compared to our study. In contrast, the 75th percentile for the fluoroscopic time was 18.1 min compared to the 24.8 min reported in our study [6].

Our findings and previous papers demonstrate a strong correlation between complexity within endovascular work with dose and fluoroscopy time [7, 28]. Fluoroscopy time, however, is not necessarily correlated with increased doses [29–31]. Although we have not implemented complexity scoring systems or performed complexity subgrouping analyses, our data support the aforementioned correlation between dose and complexity, with fixed machine analysis demonstrating the following: KAP for standard EVAR (162 Gy cm^2^), TEVAR (175 Gy cm^2^) and BEVAR/FEVAR (266 Gy cm^2^) and fluoroscopy KAP for standard EVAR (49 Gy cm^2^) BEVAR/FEVAR (190 Gy cm^2^).

Our cases only included joint IR procedures to ensure a homogenous group of operators with the same level of expertise, training and understanding of radiation protection measures. We have not analysed subgroups comparing independent vascular surgeons versus IR endovascular aneurysm repair doses, as the setup varies across all UK centres. Our study pooled data from all centres into the 3 data sets: standard EVAR, FEVAR/BEVAR and TEVAR. This study’s main limitation is the retrospective data collection, which meant that some patient demographics and variables were unavailable, such as weight and height, CAK, number of runs/acquisitions and number of angles. However, a few studies suggest DRLs can be calculated without the need to acknowledge patient weight [32, 33]. Although our data collection had limited data on weight, the study recorded all patients and is thus representative of the whole patient cohort rather than a weight-determined subset or attempts to normalise our dose metrics by a weight-derived value. Retrospective data collection also necessitated a manual review of multicentre case descriptors, allowing for unrelated cases to be identified and removed from the data set following discussions with the project leads. Similar issues have been described in numerous similar studies for DRL establishment within interventional radiology [19, 34]. Future reviews of DRL implementation and procedural doses should ideally be a prospective analysis, with automated data transfer and advancing dosimetric software to avoid human error and allow accurate data validation. A barrier to implementing this would be the difficulty arising from incorrect or inadequate system coding of procedures. All centres should develop and adopt guidance whereby coding and electronic dose-capturing systems are consistent. Although dose optimisation through the use of DRLs and the ALARA principle is paramount, this should not affect the image quality or procedural outcome.

## Conclusion

The proposed DRL for KAP was 162 Gy cm^2^, 175 Gy cm^2^ and 266 Gy cm^2^ for standard EVAR, TEVAR and BEVAR/FEVAR, respectively. This study shows that endovascular aortic procedures can be associated with significant radiation doses and that there are large inter-centre dose variations. Developing DRLs pertinent to those procedures is essential to optimise the radiation risks to patients and staff while maintaining the highest standard of patient care.
