# Correction to: National UK Survey of Radiation Doses During Endovascular Aortic Interventions

**DOI:** 10.1007/s00270-023-03632-6

**Published:** 2023-12-12

**Authors:** Yvonne Tsitsiou, Bar Velan, Rebecca Ross, Raghu Lakshminarayan, Andy Rogers, Mohamad Hamady, RADEVAIR, Lamran Khan, Ananth Krishnan, Martin Hennessy, Ram Kasthuri, Zenaib Al-Rekabi, Said Abisi, Mark Hampshire, Panos Goutzios, Muhammad Hanif, Emma Olivier, Andrew Wood, Andrew Macey, Sachin Modi, Robert Allison, Clare Bent, Peter Bungay, Robert Whiteman, Robin Williams, Zaid Aldin, Josephine Weaver, Robert Kaikini, David Wells, John Hancock, Anil Madhavan, Sapna Puppala, Matthew Matson, Katharine Lewis, Raman Uberoi, Andrew Winterbottom, Bella Huasen, Michael Jenkins, Trevor Cleveland, Rachel Butcher

**Affiliations:** 1 Imperial College Healthcare NHS Trust, St. Mary’s Hospital, Praed St, London, W2 1NY UK; 2 Department of Surgery and Cancer, Imperial College London, London, UK; 3 Hull University Teaching Hospitals NHS Trust, Hull, UK; 4 Nottingham University Hospitals NHS Trust, Nottingham, UK

**Correction to: Cardiovasc Intervent Radiol** 10.1007/s00270-023-03592-x

In the original text, in the list of RADEVAIR Collaborators (page 8) Emma Olivier’s name was misspelled as Emma Oliver.

The correct list of RADEVAIR (RAdiation Doses in EndoVascular Aortic InteRventions) Collaborators is therefore:

Lamran Khan, Ananth Krishnan, Martin Hennessy, Ram Kasthuri, Zenaib Al-Rekabi, Said Abisi, Mark Hampshire, Panos Goutzios, Muhammad Hanif, Emma Olivier, Andrew Wood, Andrew Macey, Sachin Modi, Robert Allison, Clare Bent, Peter Bungay, Robert Whiteman, Robin Williams, Zaid Aldin, Josephine Weaver, Robert Kaikini, David Wells, John Hancock, Anil Madhavan, Sapna Puppala, Matthew Matson, Katharine Lewis, Raman Uberoi, Andrew Winterbottom, Bella Huasen, Michael Jenkins, Trevor Cleveland, Rachel Butcher

The original article has been corrected.

